# Evaluating phage lytic activity: from plaque assays to single-cell technologies

**DOI:** 10.3389/fmicb.2025.1659093

**Published:** 2025-08-29

**Authors:** Vladimir Panteleev, Andrey Kulbachinskiy, Daria Gelfenbein

**Affiliations:** ^1^Laboratory of Antimicrobial Agents, Institute of Gene Biology, Russian Academy of Sciences, Moscow, Russia; ^2^Laboratory of Prokaryotic Immune Systems, Institute of Gene Biology, Russian Academy of Sciences, Moscow, Russia

**Keywords:** bacteriophages, viral infection, bacteriophage plaques, lytic cycle, single cell methods

## Abstract

Bacteriophages are the most abundant biological entities on Earth, playing critical roles in microbial ecology, evolution, and horizontal gene transfer. Since the discovery of bacteriophages in the early 20th century, a wide range of techniques has been developed to study their lytic activity. This review provides a perspective on the wide range of methods for studying phage-bacteria interactions, spanning classical bulk-culture techniques and modern single-cell and high-throughput approaches. The first section covers solid culture methods relying on plaque formation phenomenon, which allow for quantification of infectious viruses, phage host-range establishment, and analysis of certain phage traits, now augmented by robotic high-throughput screening. The second section focuses on liquid culture approaches, utilizing optical density measurements, quantitative PCR, metabolic assays and cell damage assays to measure the infection dynamics. The third section details single-cell techniques, which help to dissect the heterogeneity of infection within cell populations, using microscopy, microfluidics, next-generation sequencing, and Hi-C methods. The integration of these diverse methods has greatly advanced our understanding of the molecular mechanisms of phage infection, bacterial immunity, and facilitated phage therapy development. This review is dedicated to the 110th anniversary of phage discovery and is aimed to guide researchers in selecting optimal techniques in the fast-growing field of phage biology, phage-host interactions, bacterial immunity, and phage therapy.

## Introduction

Bacteriophages (or phages) are viruses that infect bacteria and use cell machinery for own replication. Phages were independently discovered by Twort (1915) and de Herelle (1917), who later pioneered therapeutic applications of phages for treating bacterial diseases. Twort worked with bacteria contaminating smallpox vaccines and noticed destruction areas in the bacterial colonies caused by an unknown agent, while d’Herelle observed that filtered stool extracts from dysentery patients could lyse pathogenic bacterial cultures. He identified the lytic agent as an invisible entity, naming it “bacteriophage” (from “bacterium” and Greek “phagein,” meaning “to eat”).

Phages consist of a nucleic acid genome (DNA or RNA) encapsulated in a protein coat, sometimes with a lipid envelope (Keen, 2015). Phages are the most abundant biological entities on Earth, with an estimated global population of ~10 (Strathdee et al., 2023) outnumbering bacteria by an order of magnitude (Strange et al., 2021; Batinovic et al., 2019; Mushegian, 2020). They can be found in all environments where their bacterial hosts can exist, including soil, water, and living organisms (Batinovic et al., 2019; Weinbauer, 2004). Furthermore, they can infect bacteria that live in extreme conditions such as hot springs, acid and alkaline environments, high-salinity media, and polar ice (Gil et al., 2021; Heinrichs et al., 2024; Goordial et al., 2017). Currently, the diversity and roles of bacteriophages in the microbiomes of living organisms, including humans, is actively being studied (Zhang and Wang, 2023; Townsend et al., 2021). The modern taxonomy of bacteriophages can be found on the International Committee on Taxonomy of Viruses (ICTV) website and is continuously updated (Lefkowitz et al., 2018).

Phages can be classified as virulent or temperate based on their life cycles. Virulent phages synthesize progeny and induce cell lysis shortly after infecting the host (Clokie et al., 2011), whereas temperate phages can either enter a dormant prophage state (lysogeny), coexisting with the bacterial host, or switch to the lytic cycle, producing virions and lysing the cell (Clokie et al., 2011). The choice between lytic and lysogenic cycles is tightly regulated (Clokie et al., 2011; Bruce et al., 2021; Erez et al., 2017). Some phages establish chronic infections, during which host cells continuously release viral particles without lysis (Hay and Lithgow, 2019).

Phages drove research in the “golden era” of molecular biology serving as model systems to uncover fundamental biological mechanisms (Serwer, 2025). Recent years have seen the resurgence of phages in the studies of bacterial immunity. Bacteriophages exert evolutionary pressure on susceptible bacterial strains, stimulating the development of strategies to evade or suppress infection. Through co-evolution, bacteria have evolved a diverse array of immune systems to combat viruses. These protective mechanisms include restriction-modification systems, the CRISPR-Cas system, BREX systems, Argonaute proteins, and many other known and unknown systems (Bernheim and Sorek, 2020). Infected bacteria can initiate a process known as abortive infection to protect the population from further viral replication through altruistic suicide (Lopatina et al., 2020). In turn, viruses have evolved a diverse array of anti-immune systems to counteract bacterial defenses (Georjon and Bernheim, 2023; Mayo-Muñoz et al., 2023; Gao and Feng, 2023). This host-phage arms race not only has driven the evolution of prokaryotic immune systems, but also gave rise to eukaryotic innate immunity (Georjon and Bernheim, 2023; Mayo-Muñoz et al., 2023; Gao and Feng, 2023).

Phages play diverse ecological roles. Bacterial viruses mediate horizontal gene transfer among prokaryotes, disseminating antibiotic resistance and virulence genes through the transduction process, with major medical implications (Wagner and Waldor, 2002; Fortier and Sekulovic, 2013; Chiang et al., 2019). They regulate bacterial population abundance, with estimated 15–40% of marine bacteria lysed daily by phages, impacting global nutrient availability and carbon geocycles (Keen, 2015; Wilhelm and Suttle, 1999). Phages shape the microbial community structure in various ecological niches by increasing viral pressure on dominant species, thereby promoting biodiversity (Weinbauer, 2004; Naureen et al., 2020). Notably, phage-mediated modulation of animal microbiota can influence the health of the host organism, including humans (Cryan and Dinan, 2012). Today, phage therapy is re-emerging as a promising solution to the rising threat of antibiotic resistance (Skurnik et al., 2025; Strathdee et al., 2023; Lin et al., 2017). The outcome of phage therapy critically depends on dynamic interactions between replicating phages, target bacteria, and the host immune system, and its successful development will require evaluation of detailed pharmacokinetic/pharmacodynamic parameters for phages used in treatment of specific infections (Nang et al., 2023; Hamdi et al., 2025).

The study of bacteriophages has evolved significantly over the past century, driven by advances in research methodologies. The authors recommend the review by Letarov (2020) for a detailed history of early phage research. Initial phage studies relied on basic microscopy and plaque assays to observe phage behavior and quantify their activity. The development of electron microscopy in the mid-20th century allowed scientists to visualize phage structures in detail, revealing their complex morphology. Molecular biology techniques enabled researchers to analyze phage genomes and understand their replication mechanisms.

Modern phage research spans multiple disciplines. High-throughput sequencing and bioinformatics have revolutionized this field, allowing for large-scale genomic analysis and the discovery of novel phages. Advanced imaging techniques, like cryo-electron microscopy, have provided unprecedented insights into phage structure and phage-host interactions at the molecular level. Beyond classical virology and phage molecular biology, now accelerated by cutting-edge tools, multiple studies focus on single-cell analysis of phage-host interactions.

**Figure 1:**
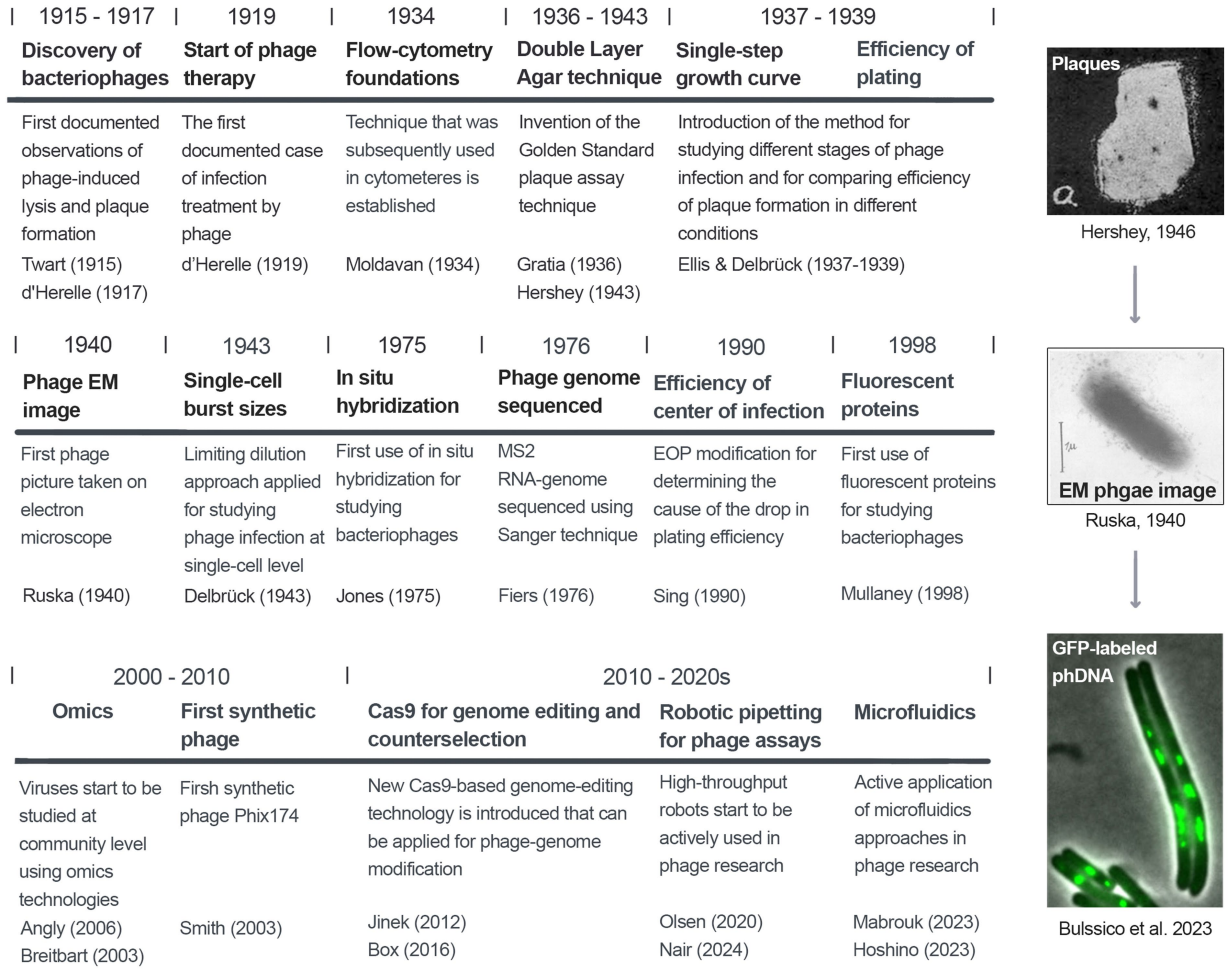
A chronological view of milestones in phage research.

We now witness a renaissance in phage research, with highly exciting strategies of phage infection and bacterial antiphage immunity discovered in recent years. Here, we systematically evaluate methods for studying the phage lytic activity, including classical and modern approaches. In particular, we cover the methods based on phage plaque formation, detection of phage lytic activities in liquid cultures, and cutting-edge approaches for studying viral infections at the single-cell level. The aim of this review is to track the evolution of phage research and assist scientists working in this field in selecting proper techniques in various fields of phage research. A short graphical introduction to the history of phage research and methodologies is shown in Figure 1. The key virological and microbiological terms used in phage research are defined in the Glossary.

## Methods to study bacteriophages in solid culture

**Figure 2:**
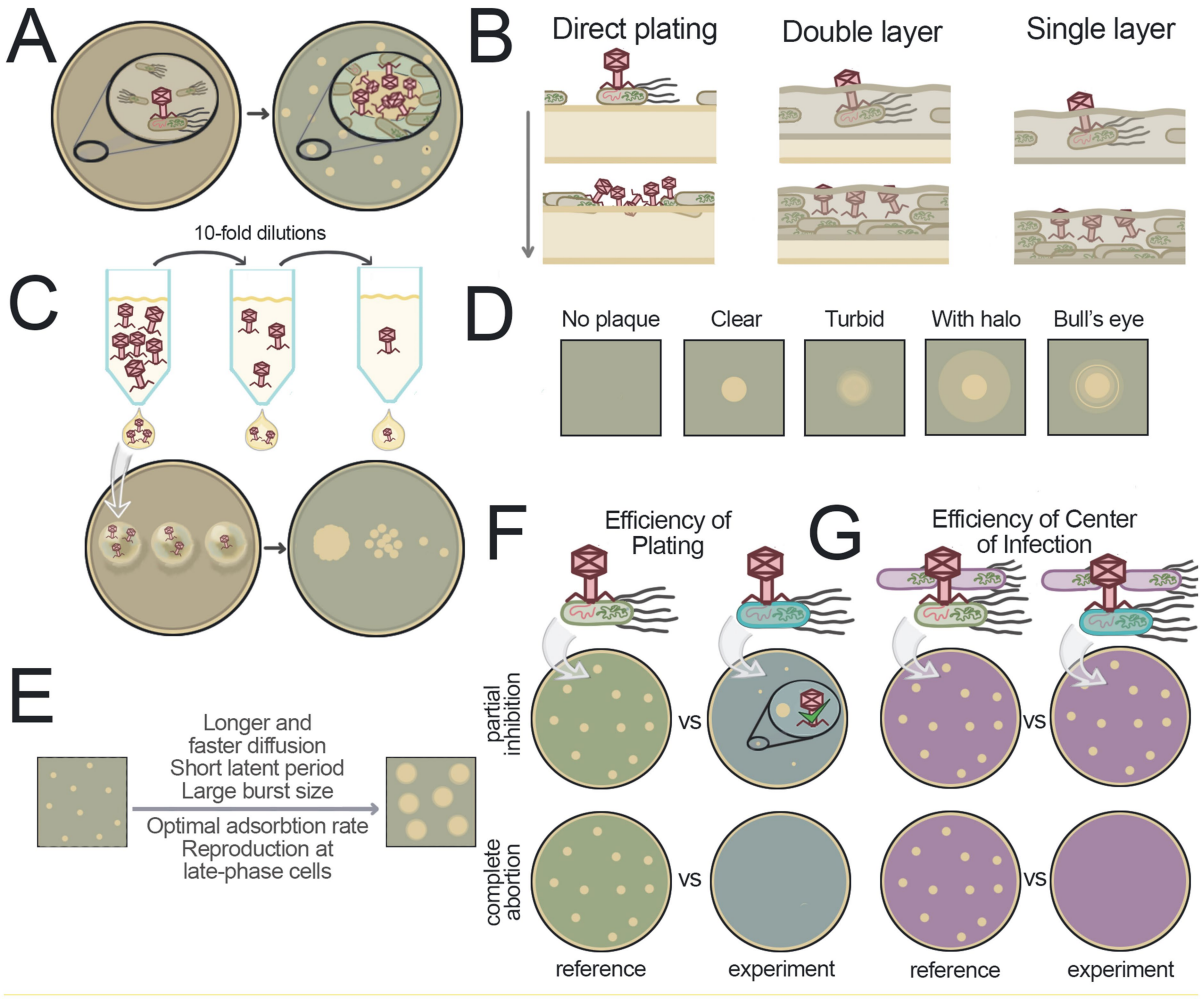
Studying phages in solid media. **(A)** Scheme of plaque formation. A zone of high phage concentration forms around the initially infected bacterium. The diffusion of virions leads to the infection of nearby bacteria, resulting in the appearance of a clear area on the agar lawn compared to the surrounding area with confluent lawn of bacteria. **(B)** Schematic representation of the positioning of bacteria and viruses in agar during plaque formation for various plaque assay methods. Yellow media represent solid agar, green media represent soft agar. **(C)** Spot test approach. Virus dilutions are applied in small volumes to the surface of solid media containing bacteria. After incubation, plaques are visible at the spots where droplets were applied, indicating successful infection. **(D)** Examples of different plaque morphologies. **(E)** Factors that contribute to the increased plaque size. **(F)** Efficiency of plating. This method measures the ability of a virus to form plaques under different conditions. In the example shown, the variable condition is the bacterial strain, green or blue. The use of the blue strain results in a reduced plaque-forming ability of the virus, which may manifest as faint plaques or the absence of plaques. **(G)** Efficiency of the center of infection. This method distinguishes between complete ineffectiveness of the viral infection and partial suppression. The strain of interest is incubated with a virus, unadsorbed particles are removed, and the cells are plated on a lawn formed by phage-sensitive bacteria (shown in purple). In the case of partial inhibition in blue bacteria, a small amount of virus will be produced, which will successfully form plaques on the purple sensitive bacteria (top). In the case of complete suppression of infection in blue bacteria, the initial viral particles will be lost and will not produce progeny, with no plaques formed (bottom).

The analysis of viral infections can be performed using either solid or liquid media, with distinct characteristics of aeration, nutrient diffusion rates, microbial distribution, and system homogeneity (Sinha et al., 2018; Somerville and Proctor, 2013). The cultures in solid (usually agar-based) media exhibit spatial heterogeneity, since viral particles diffuse more slowly through the semi-solid matrix, resulting in localized infection patterns. When a single virion infects a cell embedded in agar, its progeny can only infect nearby bacteria, leading to the formation of distinct circular zones of growth inhibition – plaques – around each initial infection site (Abedon and Yin, 2009) (Figure 2A). The groundbreaking observation was made in de Herelle (1917), who established the basis for plaque-based phage research.

This section details plaque assay methodologies, their significance in phage research, and explains how plaque characteristics can provide insights into viral behavior under varying conditions. These methods are summarized in Table 1 and Figure 2.

**Table 1:** Different plaque assay approaches.

Method	Plate features	Location of bacteria	Location of bacteriophage	Pros	Cons
Direct Plating (de Herelle, 1917; Adams, 1959)	One-layer hard agar	Agar surface	Agar surface. Application along with bacteria	Fast plate preparation: single layer agar, no bacteria inside agar	Non-homogeneous lawns; Low performance: one cell line and one phage dilution per plate
Double Agar Overlay (Gratia, 1936; Hershey et al., 1943)	Two layers: (1) Lower—hard agar, (2) Upper—soft agar	Inside agar	Inside agar. Application along with bacteria	Gold standard, perceived as the most precise technique	Low performance: one cell line and one phage dilution per plate
DLA + Spot test (Adams, 1959; Khan Mirzaei and Nilsson, 2015)			Agar surface. Application in small drops	Parallelization: many phage dilutions per plate; Easy plaques counting	Semi-quantitative results
Single Layer Agar (Havelaar and Hogeboom, 1983)	One-layer soft agar		Inside agar. Application along with bacteria	Fast plate preparation: single layer agar; Some authors claim that it gives better plaques and phage yield	Fewer plaques than in DLA; Low performance: one cell line and one phage dilution per plate
Drop cast method (Chhibber et al., 2018)	One layer. Medium agar (0.7–1.5%)	Agar surface	Agar surface. Application in small drops	Fast plate preparation: Single layer agar, no bacteria inside agar; Parallelization: many phage dilutions per plate; Easy plaques counting	Semi-quantitative results. Non-homogeneous lawns

Method	Plate features	Location of bacteria	Location of virus	Pros	Cons
Streak test (Merabishvili et al., 2009)	One-layer hard agar	Agar surface, as a streak	Agar surface in the area of streak, as small drops	Fast plate preparation: single layer agar, no bacteria inside agar; No need to count plaques; Parallelization: multiple bacteria strains and phage types per plate; Only one dilution per phage (fixed PFU for all phages)	Qualitative results
Small drop plaque assay (Mazzocco et al., 2009a)	One-layer hard agar	Bacteria and phages are premixed and applied as small drops on agar surface		Fast plate preparation: single layer agar, no bacteria inside agar; Easy to count plaques; Parallelization: multiple bacteria strains and phage types per plate	Semi-quantitative results
Robotic plaque assays (Olsen et al., 2020; Dufour et al., 2024; Nair et al., 2024)	Designs mirror other types of plaque assays involving spot test approach but robotic pipetting allows the use of smaller sample volumes			Fast: automatization, very high throughput. Parallelization: many phage dilutions per plate (in some modifications multiple bacterial strains too)	Requires expensive equipment and programming skills

### Different approaches for getting phage plaques

Standard plaque assays require three key components: (1) the virus, (2) a susceptible bacterial strain, and (3) a solid growth medium. For plaques to form, bacteria must form a confluent lawn around infectious viral particles. Two primary methods exist for preparing a bacterial lawn: (1) surface spreading of bacteria on solid agar, or (2) mixing of bacteria with molten soft agar before pouring into plates. Viruses can be introduced to the bacterial culture via: (1) pre-adsorption to bacterial cells before plating, (2) direct addition of phage to the medium, or Keen (2015) surface application of phage post-solidification.

The methods discussed in this section represent adaptations of the d’Herelle plaque assay, also known as the Spreading Method (Adams, 1959) or Direct Plating Plaque Assay (Mazzocco et al., 2009a). The Direct Plating method involves preparing an agar Petri dish, applying a mixture of bacteria and virus to its surface, and incubating the plate until plaques become visible (Figure 2B).

The most widely adopted technique, which has become the gold standard for plaque formation experiments, was independently developed by two groups of researchers in the 1940s (Gratia, 1936; Hershey et al., 1943) and is known as the Double Layer Agar (DLA) method (also called the Agar Layer method or Soft Agar Overlay) (Figure 2B). The method utilizes a two-layer agar system with the lower supporting agar serving as a source of nutrients for bacteria residing in the top soft agar. Bacteria with pre-adsorbed viruses are added to the melted soft agar and poured onto the plate with solidified lower agar. The low percentage of agar in the top layer promotes faster virion diffusion. Compared to direct plating on solid agar, DLA yields more homogeneous lawns, clearer plaques, and higher efficiency of plaque formation (Adams, 1959; Hershey et al., 1943). However, in this system bacterial physiology varies with depth – surface layers are oxygen-rich, while deeper regions become anaerobic due to limited gas exchange (Somerville and Proctor, 2013). As a result, some bacteria may switch to anaerobic metabolism. These oxygen gradients can alter phage behavior and plaque morphology.

A simplified variant of the DLA method is the Single Layer Agar (SLA) method that omits the bottom agar layer (Figure 2B). Although some studies report slightly reduced plaque counts with this approach – which can be corrected using calibration factors – SLA may enhance virion production and offers practical advantages such as faster setup, reduced resource consumption, and comparable or even superior results under certain conditions (Manikantha et al., 2022; Paranos et al., 2024; Havelaar and Hogeboom, 1983). Like DLA, SLA is widely used for environmental phage detection (Pascual-Benito et al., 2022; Fanaei et al., 2021; Korajkic et al., 2021; Cho et al., 2018; McMinn et al., 2018; Rodríguez et al., 2012).

To make the plaque assay faster, small volumes of viral suspensions can be applied to the surface of a plate as droplets. This approach, known as the Spot Test, allows for parallel testing of multiple phages stocks and phage dilutions on a single plate (Figure 2C) (Khan Mirzaei and Nilsson, 2015).

The Drop Cast method combines elements of the Direct Plating plaque assay and the Spot Test approach. Similar to Direct Plating, bacteria are not inoculated into agar but are applied to a single-layer hard-agar plate using a flooding technique (Chhibber et al., 2018). Phage dilutions are then added to the surface as droplets. A related technique, called the Small-Drop plaque assay (Mazzocco et al., 2009b) uses small volumes of both virus and bacteria, which are mixed together and applied to the surface of solid agar in droplets. After incubation, small circular lawns with plaques appear on the agar surface. A major advantage of the Small-Drop assay is that it allows testing multiple bacterial strains and different viruses on a single plate.

The use of robots can significantly accelerate the experiments relying on plaque formation but the need for expensive equipment makes robotic methods rarely used compared to manual techniques. Such methods are generally similar to the Spot Test approach but allow for smaller volumes and larger number of samples as robotic dispensing is much more precise. Published methods differ in the protocol specifics and the level of miniaturization. In the study by Olsen et al. (2020), a classic combination of DLA and Spot Test is used: small volumes of virus are applied to the surface of soft agar with bacteria on a square plate, with volumes not exceeding 1 μL, totaling 96 phage dilutions per plate. The method of Dufour et al. (2024) resembles a high-throughput Drop Cast technique: bacteria are applied to the surface of a square agar plate using flooding, and robot applies 5 μL virus samples on the surface. This also allows for the application of up to 96 samples per plate. In comparison, a high-throughput Micro-Plaque Assay employs robotic pinning platform with a capacity of 1,536 samples per plate (Nair et al., 2024). In this method, 100 nL bacterial samples are applied to a plate with nutrient agar, followed by an equal volume of viral samples deposited by a robot. Similar to the Small-Drop plaque assay, small circular lawns are formed (though much smaller in size) after a short incubation period. Currently, this is the fastest version of the plaque assay for large-scale studies.

Phage DisCo (Phage Discovery by Co-Culture) (Rand et al., 2025) is an advanced adaptation of classical solid-medium techniques. It employs multiple bacterial strains, each labeled with distinct fluorescent markers, within a single sample to investigate receptors, phage defense systems, and other cellular components critical to infection dynamics. Formation of a clear plaque indicates that all co-cultured strains are susceptible to lysis by the studied phages. Conversely, colored plaques indicate resistance of the corresponding fluorescently tagged strain to infection. This approach enables rapid, parallel screening of host-range specificity in mixed samples.

### Factors affecting plaque morphology

Analysis of the plaque morphology can be used to evaluate the lytic activity of phages. Distinct clear plaques reflect potent lytic activity, while turbid plaques reflect incomplete bacterial lysis and may indicate suboptimal virus infectivity (van Charante et al., 2019; Yin, 2008).

Historically, researchers described morphological characteristics of plaques using tools like rulers and micrometers (Reddy et al., 1982). Today, the standard approach involves high-resolution plate imaging followed by digital analysis. Software like Fiji (Schindelin et al., 2012) enables manual plaque measurements but the results remain subjective, especially for small turbid plaques. Programs like Plaque Size Tool (Trofimova and Jaschke, 2021), Viral Plaque (Cacciabue et al., 2019) automate plaque counting, sizing, and titer calculations. However, automatic tools still exhibit lower precision with turbid plaques and require manual image preprocessing.

Figure 2D illustrates several common plaque types:

1. The absence of plaques signifies very low phage activity.
2. Clear, large plaques with sharp edges signify robust lytic activity.
3. Turbid plaques reflect compromised lytic efficiency.
4. Plaques with halos (bacterial growth inhibition zones) often imply exopolysaccharide depolymerase activity, which some phages use to expose cell-surface receptors (Knecht et al., 2020).
5. “Bull’s eye” plaques with clear center and turbid periphery suggest more efficient replication during early stages of lawn development.

Even closely related phages can produce plaques with striking morphological diversity, influenced by the biology of both viruses and bacteria, as well as the growth conditions. The limiting factor in the growth of plaques is the inability of most viruses to replicate productively in bacteria during the later stages of lawn development in nutrient-depleted environments (Pires et al., 2021; Łoś et al., 2007). At this point, bacteria typically enter the stationary growth phase, their most common state in natural habitats (Koskella et al., 2022). Plaque formation process can therefore be viewed as a race between bacteria and phages, where the virus must form a plaque before the cells lose their sensitivity (Abedon, 2021). However, some phages can exploit stationary-phase bacteria, hijacking cellular metabolism despite resource limitations (Woods, 1976; Silva et al., 2024). For example, T7 bacteriophage forms unusually large plaques mainly because it can productively replicate on host cells at late stages of lawn development (Xu et al., 2021; Yin, 1991).

The size variations of plaques produced by different phages or by the same virus under varying conditions depend on several interrelated factors (Figure 2E): (1) latent period duration, (2) burst size, (3) virion diffusion rate, and (4) adsorption efficiency (Abedon and Yin, 2009). While shorter latent periods allow more time for diffusion, they typically reduce burst size. Conversely, longer latency increases burst size but leaves less time for virion spread. Although a larger burst size contributes to increased plaque size, its impact is less significant than that of a shortened latent period (Abedon and Yin, 2009; Abedon and Culler, 2007). Diffusion dynamics also plays a critical role: jumbo phages (>200 kb genomes) move more slowly through agar, often forming only tiny plaques (Yuan and Gao, 2017). Lowering agar concentration enhances diffusion, making giant phage plaques more readily detectable (Saad et al., 2019; Kwon et al., 2020) and generally increasing plaque size for other viruses. Adsorption rates also affect plaque expansion by modulating viral diffusion. Moderate or low adsorption allows wider virion spread, while high adsorption confines infection to nearby cells (Abedon and Yin, 2009).

Several additional factors can affect the clarity, size and other morphological characteristics of plaques, even leading to their complete disappearance. These factors include: (1) composition of growth media (Ramesh et al., 2019; Anderson, 1948a), (2) supplements such as glycerol (Türe et al., 2022; Santos et al., 2009; Jamasbi and Paulissen, 1978) or bile acids (Jamasbi and Paulissen, 1978), (3) the presence of adsorption-promoting cofactors like bivalent metal salts (Cvirkaitė-Krupovič et al., 2010; Harada et al., 2013; Mikolajcik, 1964) or L-tryptophan (Anderson, 1948b), and (4) temperature (Anderson, 1948a). Additionally, oxygen availability significantly affects bacterial physiology and may influence viral infection (Hernández Villamizar et al., 2023). Anaerobic conditions can both enlarge plaques or induce edge turbidity, depending on the phage and bacteria type (Hernández Villamizar et al., 2023; McConnell and Wright, 1975).

Under certain conditions, zones of bacterial growth inhibition or partial lysis can be observed at low phage dilutions (high phage titers) with no plaque formation at higher dilutions. This phenotype may result from failure of individual viral infections and inability of single viral particles to form progeny, despite their ability to infect cells. At low phage dilutions, unproductive infections of bacterial cells by numerous viral particles may result in zones of bacterial growth suppression. This phenomenon is known as Lysis From Without, or Non-Productive Lysis – premature cell lysis caused by massive phage adsorption and cell death through fatal membrane damage and/or metabolism alterations without productive infection (Abedon, 2011).

Temperate viruses often fail to form plaques or produce only turbid ones under standard conditions because the majority of phages may enter a prophage state without lysis of the infected cells (Makky et al., 2021; Brady et al., 2021; Oppenheim et al., 2005). Stress conditions can force temperate phages to enter the lytic cycle, resulting in clear, well-defined plaques (Islam et al., 2012). Thus, prophage inducers like mitomycin, which triggers the SOS response via DNA damage, are employed to enhance the visibility of plaques (Islam et al., 2012). Some temperate phages naturally exhibit sufficient lytic-cycle entry and form plaques even without induction (Bertani, 1951). Notably, subinhibitory concentrations of antibiotics can sharpen plaques for both temperate and virulent phages by modulating bacterial physiology (Santos et al., 2009; Loś et al., 2008; Kaur et al., 2012). Table 2 provides a summary of tips for working with various types of phages in solid media.

**Table 2:** Plaque assay recommendations for several common phage types.

Phage type	Requirements for successful plaque formation	References
Highly lytic	Standard plaquing procedure	Gratia (1936) and Hershey et al. (1943)
Temperate	Typically, faint or no plaques are formed under standard conditions. Prophage inducers, such as mitomycin C or other antibiotics, can be used to enhance plaque formation. Alternatively, adding glycine to the agar or using low-density top agar can improve results.	Lillehaug (1997) and Loś et al. (2008)
Jumbo phages	Typically, faint plaques or no plaques are formed under standard conditions. A DLA plaque assay with a very low density top agar ( ≤ 0.35%) and reduced incubation temperatures can be used to enhance plaque visibility.	Saad et al. (2019)
F-specific phages	Plaque formation should be done with male bacterial strains because these phages require F-pili for adsorption.	Hata et al. (2016)
Phages establishing chronic infection	Infected cells remain viable, but their generation times are prolonged, resulting in slower-growing bacterial plaques. Due to the transient nature of these plaques, shorter incubation periods are recommended.	Green and Sambrook (2017)

Real-time monitoring of plaque formation can help to reveal dynamic changes in the plaque morphology. It can be achieved by manually photographing the plates (Koch, 1964) or using automated photographing systems (Perlemoine et al., 2021; Lee and Yin, 1996). Real-time monitoring is especially useful for phages with temporary plaques (e.g., filamentous phages) that become overgrown with bacterial lawn over time.

In summary, the plaque morphology is highly dynamic and depends on the biological properties of the virus, the physiological traits of the bacteria, and external factors, and can therefore serve only as a rough indicator when describing the biology and behavior of the virus.

### Using plaques to estimate the efficiency of phage infection

Plaque assays enable qualitative and quantitative cross-condition comparisons of phage activity. Qualitative methods are used for preliminary characterization of the virus infectivity. Spot Test is often used for initial qualitative testing, with a few or just a single phage dilution (Khan Mirzaei and Nilsson, 2015). Streak Test is another qualitative method, in which bacteria are applied onto the surface of solid agar as narrow streaks and phages, taken at a fixed titer, are applied as small drops at equal intervals along the streak. Following incubation, bacterial streaks appear on the plate with plaques or lysis areas at the spots of phage application (Merabishvili et al., 2009). This allows to test different bacterial strains and phages on a single plate (Merabishvili et al., 2009; Cooper et al., 2011). However, qualitative tests provide only rough estimate of the phage infectivity (Yes or No) and cannot distinguish productive and unproductive infections.

The golden standard for quantitative assessment of the viral replication efficiency and infectivity is the method of relative Efficiency Of Plating (EOP), first introduced in 1939 (Figure 2F) (Ellis and Delbrück, 1939). The relative EOP is a ratio of the number of plaques (Plaque Forming Units, PFU/ml) in the experimental sample under specific conditions to the number of plaques measured in reference conditions (e.g., using a reference phage-sensitive bacterial strain). When calculating the relative EOP, the spot test approach is often used for scalability, allowing for quick assessment of the host range and various conditions (Khan Mirzaei and Nilsson, 2015). In comparison, the absolute EOP corresponds to the number of infectious viruses determined in the plaque assay under given conditions relative to the absolute number of viral particles (Kinnunen, 1978). However, calculation of the absolute EOP is resource-intensive, requires using of sophisticated techniques like electron microscopy, and is rarely applied.

Importantly, changes in the PFU numbers in the relative EOP assay under specific experimental conditions may be due to either reduced viral replication efficiency (decreased burst size) or complete inhibition of virion production in infected cells in these conditions. In the first case, some plaques may still form but may not be visible due to their small size, while in the second case no plaques form at all. A method called the Efficiency of Center Of Infection (ECOI) was developed to distinguish between these scenarios (Figure 2G) (Sing and Klaenhammer, 1990). In the first step of the experiment, the cells of interest and control cells are infected with the bacteriophage, forming “infection centers.” The infected cells are then washed to remove free virus and a standard plaque assay is carried out with the cell lawn formed by phage-sensitive bacteria. This ensures that if even a small amount of virus is produced in the initially infected cell, a plaque will form on the lawn formed by virus-sensitive bacteria. On the other hand, the absence of plaques will indicate that the phage does not produce progeny in the initially infected bacteria.

In summary, methods relying on plaque formation are relatively simple and accessible, require minimal specialized equipment, and allow to explore different aspects of phage biology and perform quantitative analysis of phage titers and infectivity. These assays also make possible the isolation of individual phage clones from complex mixtures, a classic approach that can hardly be replaced even by modern techniques. However, these methods can be labor-intensive and time-consuming due to long lawn formation times, the need for plaque quantification and morphological analysis, and cannot be readily applied to all bacteria and bacteriophages since not all bacteria form lawns and not all phages form clear plaques. Notably, plaque counts only approximate viral particle numbers, as various factors may reduce or increase the viral replication and infectivity. Nevertheless, the plaque formation phenomenon remains one of the most important parts of phage research.

## Methods to study bacteriophages in liquid culture

**Figure 3:**
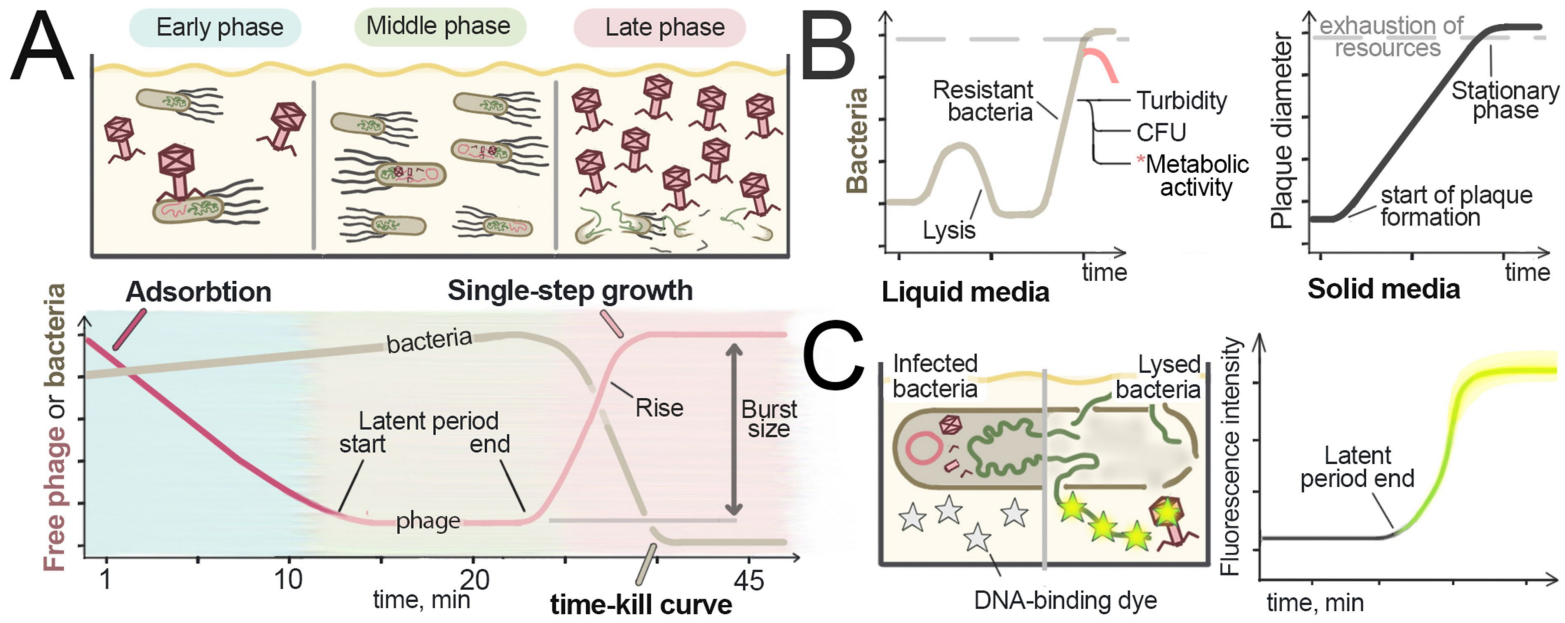
Studying phages in liquid culture. **(A)** Measurement of the adsorption rate, latent period, and burst size by assessing free virus in liquid culture. The amounts of free virus and bacteria in the culture at different steps of infection are schematically shown at the top. An example of the single-step growth curve is shown at the bottom. To determine the amount of free virus in the culture during different steps of infection, bacteria are removed from samples and the viral quantity is measured (e.g., using the plaque assay). To establish the rate constant of adsorption, the viral quantity is measured immediately after the virus is added, and the rate of viral titer decline over time is recorded. For assessing the latent period and burst size, samples are taken from the culture after the adsorption period in synchronously infected cultures. The increase of free virus amounts indicates the end of the latent period. The difference in the amount of free virus during the latent period and after its release from infected cells corresponds to the burst size. The end of the latent period and the appearance of new viral particles leads to bacterial lysis, which is manifested in a OD decrease. **(B)** Comparison of the virus activity in solid and liquid media. In solid media (left), plaque formation is a linear process that continues until nutrients are depleted [adapted from Abedon, 2021]. In liquid media (right), the progression of viral infection is nonlinear; after lysis, the remaining resistant bacteria can repopulate the culture. The pink line at the end of the graph represents the decline in the metabolic activity of bacteria in the stationary phase. **(C)** Detection of bacterial lysis using DNA-binding fluorescent dyes that cannot penetrate intact cell membranes (grey stars). DNA and viral particles are released from the cells during lysis, resulting in an increase in fluorescence, showed in yellow stars. The graph corresponds to fluorescence intensity measured over the time.

Bacteriophages are capable of infecting bacteria in liquid environments, resulting in either complete or partial lysis of the bacterial culture. In contrast to plate-based assays, bacteria and viruses are uniformly distributed in liquid cultures, allowing the entire volume to be treated as a single homogeneous system. Under these conditions, successful phage replication typically leads to complete bacterial lysis as all cells become infected. Visualizing infection and collecting samples in liquid cultures is generally easier than in solid media. Liquid culture assays are an effective alternative to solid-media methods for determination of the phage host range, replication efficiency, and lytic activity (Doss et al., 2022). This section covers the methods used to study phage infections in liquid media, summarized in Figure 3 and Table 3.

**Table 3:** Methods for measuring phage lytic activity in liquid culture.

Measured parameter	Means of measurement	The essence of the method	Special materials/equipment	Pros	Cons
Culture Turbidity	Eye (de Herelle, 1917)	Lysed cultures are less transparent which allows observation of the drop in culture turbidity upon lysis	None	Simple routine approach often employed for phage propagation; Minimal equipment requirements	Qualitative and highly subjective
	Optical Density (Doss et al., 2022; Rajnovic et al., 2019)	Phage-lysed bacteria possess decreased light-scattering properties. Decrease in light-scattering can be measured by spectrophotometers	Spectrophotometer	The most frequently used approach for monitoring phage infection; Easy to scale: compatibility with plate readers and robotic pipetting; Easy real-time monitoring; Minimal equipment requirements	Overestimation of bacterial counts: lysed and metabolically inactive bacteria can still scatter light
Free phage (Kropinski, 2018; Adams, 1959; Kropinski, 2009; Ellis and Delbrück, 1939)	Plaque assay	Phage adsorption and successful phage infection lead to changes in culture PFU values which can be monitored using plaque assay	Conventional materials for plaque assay experiments	Gold standard for studying phage growth parameters; Minimal equipment requirements	Difficult to scale: requires the collection of multiple samples and subsequent plaque assay experiments
Viable cell number (Davey, 2011)	Cell plating	Single bacterial cells can form visible colonies, which can be used to calculate the decrease in the number of viable cells in the culture upon lysis	Conventional microbiological equipment for CFU measurements	The most common approach for measuring viable cell counts; Minimal equipment requirements	Difficult to scale: requires collecting multiple samples and subsequent cell plating experiments; Underestimation of cell numbers: phage infected and dormant cells often fail to form colonies
Cell metabolic activity	Reduction of tetrazolium salts or their analogues (Smith and McFeters, 1997; Henry et al., 2012)	Tetrazolium salts are reduced to colored compounds by cell RedOx activity which drops upon phage lysis	Materials for tetrazolium-based viability assay, spectrophotometer	Easy real-time monitoring; Easy to scale: compatibility with plate readers and robotic pipetting	Depends on the accumulation of colored products over time: later changes in culture can be difficult to detect, and lytic events are not detected instantly
	CO_2_ release (Eaton, 1931; Sauvageau et al., 2010)	Viable and metabolically active cells release CO_2_. Phage lysis stops metabolism and CO_2_ generation	Equipment for measuring CO_2_ levels, such as IR-spectrometer with CO_2_ sensor.	Easy real-time monitoring when using IR-spectrometry	Difficult to scale: no multi-sample solutions available; Only applicable for relatively big-volume cultures; Dormant cells can be difficult to detect
Cell metabolic activity	Oxygen Uptake Rate (Daugelavičius et al., 2024; Werquin et al., 1984)	Viable cells consume oxygen during respiration. Phage lysis stops respiration processes	Equipment for measuring O_2_ levels, such as dissolved oxygen probe.	Easy real-time monitoring when using dissolved oxygen probe	Difficult to scale: no multi-sample solutions available; Only applicable for relatively big-volume cultures; Dormant cells can be difficult to detect
	Heat Production (Maskow et al., 2010; Braissant et al., 2010)	Heat production is a feature of metabolically active organisms. Phage lysis stops heat production	Microcalorimeter	Easy real-time monitoring	Difficult to scale: no multi-sample solutions available; Only applicable for relatively big-volume cultures; Dormant cells can be difficult to detect
	Lux system activity (Meighen, 1991; Egido et al., 2023)	Constitutively expressed Lux system generates luminescence signal. Phage lysis stops luminescent signal generation	Luminometer	Easy real-time monitoring; Easy to scale: compatibility with plate readers and robotic pipetting	Requires special bacterial strains expressing the Lux system
Loss of membrane integrity	Binding of membrane-impermeable dyes to exposed cell DNA (Egido et al., 2023)	Loss of membrane integrity upon phage lysis allows membrane impermeable dyes to enter the cell and bind DNA	DNA-binding fluorescent dyes that cannot permeate cellular membranes (such as Sytox9)	Easy real-time monitoring; Easy to scale: compatibility with plate readers and robotic pipetting	Difficult to apply for phages, degrading host DNA
Loss of membrane integrity	ATP and enzyme release (Blasco et al., 1998; Squirrell et al., 2002)	Detection of the increase in ATP concentration or specific enzymatic activity (such as NADH production) in culture medium upon lysis	Reagents for luciferase-based ATP detection assays and luminometer	Detection of lytic processes on a miniaturized scale: detecting enzyme release is ultra-sensitive due to enzyme multi-round nature	Difficult to scale: requires the collection of multiple samples and subsequent metabolite/enzyme detection
	Two-dye viability assay (Latka and Drulis-Kawa, 2020; Decker, 2001)	Two DNA-binding dyes are used, one of which can permeate cell membranes, while the other can only penetrate dead cells with damaged membranes. Phage lysis leads to increase in dead-stained cells numbers	Two dyes appropriate for the assay (or kit, such as LIVE/DEAD BacLight Bacterial Viability Kit), fluorimeter	Compatible with fluorescence microscopy and flow cytometry	Difficult to scale: requires the collection of multiple samples and subsequent staining; Harder to apply for phages, degrading host DNA; Need for calibration curves
	Detection of changes in the ionic composition of the medium (Ziedaite et al., 2005)	The ionic composition of bacterial cytoplasm is different from the environment. The sharp increase in the concentration of certain ions (e.g., K+) upon cell lysis can be detected	Ion-selective electrode	Easy real-time monitoring when using electrodes	Difficult to scale: no multi-sample solutions available; Usually applied for relatively big-volume cultures
Loss of membrane integrity	Membrane depolarization detection (Daugelavičius et al., 2024; Ziedaite et al., 2005)	Depolarization events, accompanying lytic processes, cause the accumulation (e.g., PCB-) or release (e.g., TPP+) of certain compounds on/from the cellular membrane. Measuring their free/cell bound concentration ratio allows to monitor lysis	Special compounds (e.g., TPP + or PCB-), selective electrodes	Allows to detect membrane structure changes which start earlier than other lysis events; Easy real-time monitoring when using electrodes	Difficult to scale: no multi-sample solutions available; Usually applied for relatively big-volume cultures
Cellular and viral DNA abundance (Abbott et al., 1988; Morella et al., 2018)	qPCR	Changes in cell and viral DNA abundance can be monitored using qPCR, giving data on phage infection progression	Real-Time PCR Instrument, primers and reagents	Estimates viral and bacterial DNA dynamics; Viral DNA abundance can be used for calculation of PFU using calibration curves; Some qPCR modifications allow more precise measurement of only infectious phage DNA and estimation of its degradation	Difficult to scale: requires the collection of multiple samples, sample preparation and design of qPCR experiments.

### Kinetic assays of phage infection

Changes in the cell density during infection are the most evident manifestation of phage activity in liquid culture, which can be detected by either end-point or kinetic measurements. Early researchers relied on visual assessment of culture densities for monitoring the progression of phage infection (de Herelle, 1917), a method still in use for qualitative analysis (Bonilla et al., 2016). Quantitative culture turbidity (or optical density, OD) measurements, usually performed by spectrophotometers, are now widely used to study viral infection and cell lysis in liquid media (Figure 3A). Plate readers can be employed for high-throughput sample processing, making possible parallel analysis of multiple samples. A recent study employed a 384-well plate high-throughput turbidimetric assay for determination of the host range of newly isolated bacteriophages. This approach enabled simultaneous testing of four phages against 22 bacterial strains with technical triplicates in a single assay (Martinez-Soto et al., 2021).

Kinetic assays of cell growth are generally preferred over endpoint OD measurements since they allow to record different stages of phage infection and build growth curves showing lysis kinetics and culture collapse (“time-kill curves”) (Figure 3A, bottom) (Doss et al., 2022). Since the outcome of phage infection in a liquid culture greatly depends on the ratio of phage particles to host bacteria, the multiplicity of infection (MOI) parameter is usually controlled and recorded to describe infection conditions. At low MOI, when initial virus titers are much lower than the number of cells in the population, multiple infection cycles can occur, with newly produced virions initiating secondary infections before the final culture lysis. At high viral loads, complete culture lysis and virus release occur rapidly and often before nutrient depletion allowing subsequent re-growth of resistant bacteria (Figure 3B, left graph; compare with cell growth in solid media, right graph). Recent work (Geng et al., 2024) showed that the lysis timepoint changes linearly with logarithm of phage concentration, enabling calibration curve-based titer determination under standardized conditions.

Notably, viruses and antibiotics produce similar growth curve patterns. The Minimal Inhibitory Concentration (MIC) concept, representing the lowest antibiotic concentration inhibiting bacterial growth, can be applied to bacteriophages by defining the minimal MOI required to inhibit bacterial proliferation (Kowalska-Krochmal and Dudek-Wicher, 2021; Phillips et al., 1998).

Comparative bacterial growth curve analysis enables assessment of the viral activity through: (1) visual evaluation of the lysis timing/degree, which is subjective and prone to biases, (2) by using computational growth curve metrics. In the second approach each curve is assigned a lysis efficiency score based on its lysis pattern, enabling quantitative comparison between curves. Multiple mathematical methods exist for computational curve analysis, differing in their method of calculation of lysis efficiency. These methods use such metrics as Lysis Score (Bourdin et al., 2014), Virulence Index (Storms et al., 2020), Phage Score (Konopacki et al., 2020), and Centroid of the Bacterial Growth Curves (Hosseini et al., 2024).

### Phage adsorption rate, latent period, and burst size

Typical phage infection features three phases: (1) Early phase – viral adsorption and initiation of infection, (2) Middle phase – bacterial growth with intracellular phage replication, and (3) Late phase – culture collapse through lysis (Figure 3A, upper scheme). These phases are accompanied by changes in the free phage concentration in the medium: (1) Early phase – free phage concentration decreases due to adsorption; (2) Middle phase—stable free phage concentration; (3) Late phase—phage progeny release and increase in free phage concentration. Tracking free virus concentration can thus be used to reveal transitions between these stages, and calculate the adsorption rate, latent period duration, and the burst size (the number of virions produced per infected bacterium) (Figure 3A, lower graph).

Outside the cell, the virus exists in an inactive state termed ‘extracellular search” (Hyman and Abedon, 2009), during which it diffuses randomly until encountering a susceptible bacterium. The ability of a virus to attach to the target cell is a crucial factor determining its host range (Adams, 1959), which can be expanded or restricted by altering the virus specificity to cell surface epitopes (Zhang et al., 2022).

The adsorption rate constant can be determined by monitoring virion concentration decline in the medium during early phases of infection. In this approach (Adams, 1959), bacteria and phage are mixed, and free virions are quantified at short intervals via plaque assay. To halt viral replication in sampled bacteria, the cells may be removed by chloroform treatment or centrifugation (Sechaud and Kellenberger, 1956). Adsorption kinetics is derived by plotting normalized viral titers against time (Figure 3A, lower graph). Viral adsorption can be described as a bimolecular reaction between viral and bacterial components, characterized by standard isothermal adsorption equations (Krueger, 1931). Viral adsorption is accelerated at high concentrations of both the phages and bacteria (Ellis and Delbrück, 1939; Krueger, 1931; Schlesinger, 1932). Elevated phage concentrations also increase the probability of superinfection of a single bacterium with multiple phages (Krueger, 1931; Burnet et al., 1938), which in some systems triggers “lysis from without” (Abedon, 2011). Importantly, bacterial cell size also influences phage adsorption, with the adsorption rate increasing as the cell surface becomes bigger (Schlesinger, 1932; Abedon, 2023).

The latent period and burst size can be determined in a Single-Step Growth assay, first described in 1939 (Ellis and Delbrück, 1939). A known amount of phage is added to a log-phase bacterial culture, at MOI < 1 to avoid superinfection, followed by viral titer measurements at regularly spaced intervals. Plotting the resulting titers versus time reveals a steep increase corresponding to synchronous release of virions from lysed cells. This single-step growth curve (Figure 3A, lower graph) captures one infection cycle. The time between the end of viral adsorption and the onset of viral release from cells corresponds to the population average latent period (Abedon et al., 2003).

The burst size can be calculated from free virus titration at the following points: (1) initial virus added; (2) virus remaining after adsorption; (3) virus immediately preceding lysis; (4) virus after lysis completion. The burst size is determined as the ratio of the progeny viral particles [PFU after lysis (4) minus PFU before lysis (3)] to the adsorbed viral particles [PFU added (1) minus PFU after adsorption (2)].

To obtain accurate calculation of these parameters, synchronized infection is required to ensure nearly simultaneous initiation of infection and virion release across the bacterial population (Adams, 1959; Eisenstark, 1967). Highlighting the importance of synchronized conditions, Eisenstark (1967) compared single-step growth experiment to a horse race, where phages must be aligned at the starting line in order to get reliable measurements. Uniform initiation of infection can be achieved by viral adsorption in nutrient-depleted medium with subsequent transfer to nutrient-rich medium that initiates synchronized viral replication (Eisenstark, 1967). However, when using high-density bacterial culture, >90% viral adsorption occurs within minutes, making timing variations negligible in many cases (Krueger, 1931).

Non-synchronized and secondary infections can also be prevented by: (1) culture dilution (50-100-fold) to reduce phage-bacterium encounter probability, (2) removal of free phages after adsorption step by centrifugation, and (3) replacement of the growth medium to deplete cofactors required for phage adsorption (Adams, 1959; Kropinski, 2009; Ellis and Delbrück, 1939).

Some microbiological techniques allow to measure phage burst size and its variation among individual cells, by diluting and placing infected cells into separate test tubes until there is only one or fewer bacteria per tube (Delbrück, 1945). This approach allows to calculate the variation in the number of viruses per cell by measuring phage titer in each tube after lysis (Delbrück, 1945), and can be used to find differences among cell subpopulations (Kannoly et al., 2022).

### Detection of live bacteria and metabolic activity

The number of live bacterial cells during infection changes dramatically and can be directly determined by measuring the number of colony-forming units (CFU) in the culture. In comparison with the turbidity (OD) measurements, which also detect non-dividing and dead cells and cell debris, CFU counting allows accurate detection of only viable cells that can divide and form single colonies on solid media (Pla et al., 2015; Davey, 2011). CFU counting can be used either in an endpoint format to assess the effectiveness of viral elimination of bacteria (Roger, 1977; Latka and Drulis-Kawa, 2020) or in a kinetic format to construct cell lysis curves (Eaton, 1931; Melo et al., 2018). CFU measurements can be used for bacterial cell counting not only in liquid cultures but also in biofilms, for example to evaluate the efficiency of bacterial elimination phage depolymerases and antibiotics (Latka and Drulis-Kawa, 2020). It should be noted that direct CFU measurements in the infected culture may result in underestimation of the true bacteria count at the time of sampling due to the ongoing phage activity and progressive infection after plating (Verthé and Verstraete, 2006). To overcome this complication, the OD values of the infected bacterial culture, measured in real-time, can be converted to CFU using calibration curves (Hernández Villamizar et al., 2023; Sezonov et al., 2007).

CFU measurement is a cheap and straightforward method that requires only basic microbiological equipment and reagents, but the procedure is time-consuming, involving sampling, plating, and waiting period for colonies to form. It also does not allow to identify bacteria that are in a dormant state and retain metabolic activity but are unable to form colonies (Davey, 2011).

As an alternative, direct measurements of the bacterial metabolic activity can be used to detect the phage lytic activity. It is important to note that the level of metabolic activity does not always correlate with bacterial biomass. For example, in the stationary phase, high bacterial numbers correlate with high OD and CFU, but their metabolic activity may be low (Braissant et al., 2020). However, phage-induced cell lysis usually results in a sharp decrease in the metabolic activity (Figure 3B, left graph). The metabolic activity can be estimated by measuring the concentrations of ATP and cellular metabolites, redox activity, or monitoring other physiological changes in infected cells in real time (Ralston and Baer, 1963). For detailed protocols and interpretation, see reviews by Braissant et al. (2020) and Davey (2011).

Intracellular ATP concentration varies depending on environmental conditions (Deng et al., 2021) and reflects bacterial viability (Schneider and Gourse, 2004). The ATP level is generally proportional to the number of live bacteria and can thus be used to detect the phage lytic activity (Daugelavičius et al., 2024; Santos et al., 2024; Ziedaite et al., 2005). To quantify intracellular ATP, the cells are lysed and released ATP is measured by firefly luciferase (Thorne et al., 2010) or horseradish peroxidase (Veitch, 2004). Importantly, extracellular ATP released during phage-induced cell lysis must be removed for accurate biomass estimates.

The RedOx activity also reflects cell viability and can be assessed using tetrazolium salts. In the oxidized form, these salts are colorless but can be reduced to colored formazan derivatives by the enzymes of bacterial respiratory chain (Smith and McFeters, 1997). Phage-induced cell lysis can thus be detected as a drop in this activity. In addition to the ability to detect the elimination of bacteria by viruses in individual experiments (Dalmasso et al., 2015; Sanchez et al., 2022; Lin et al., 1977), high-throughput Phage Susceptibility Testing (PSA) using the same principle has been developed for robotic platforms like OmniLog^™^ for analysis of phage replication, phage host range, and (Henry et al., 2012; Cunningham et al., 2022). In one of the studies, the OmniLog^™^ platform allowed testing of 19 *E. coli* phages against 18 bacterial isolates, and 21 *Staphylococcus aureus* phages against 11 bacterial isolates (Cunningham et al., 2022). A similar principle is applied in resazurin-based viability assays, which were successfully employed to detect the lytic activity of bacteriophages and increased cell survival conferred by defense systems (Meeske et al., 2019).

During respiration, bacteria release carbon dioxide, which can serve as an indicator of their metabolism during phage infection. Early phage studies relied on KOH for absorption of CO_2_ from cell cultures (Eaton, 1931; Warburg, 1926). Modern infrared spectroscopy enables real-time CO_2_ monitoring in infected cultures (Sauvageau et al., 2010). For example, this method was applied to study T4 phage infection, allowing not only the observation of phage-induced lysis but also the detection of specific events during the infection process (growth rate, rate or respiration and its derivatives) (Sauvageau et al., 2010). On the other hand, an increase in bacterial oxygen uptake rate (OUR) can indicate both an increase in bacterial mass and ongoing lysis. OUR can be measured in real time using soluble oxygen probes (Daugelavičius et al., 2024; Werquin et al., 1984; Maskow et al., 2010). For example, measurements of dissolved oxygen were used to assess the efficiency of phage production in fermenter cultures of *Rhizobium meliloti* (Werquin et al., 1984). Heat production can also be measured as a proxy of bacterial metabolism, using isothermal microcalorimetry that allows highly sensitive detection of heat changes during culture growth and phage-induced lysis (Maskow et al., 2010; Tkhilaishvili et al., 2018; Guosheng et al., 2003; Braissant et al., 2010). For instance, calorimetry allows real-time monitoring of lambda prophage induction (Maskow et al., 2010) as well as noninvasive assessment of bacterial eradication efficiency in biofilms (Kunisch et al., 2024).

Bacterial luciferase (the luxCDABE system from the bioluminescent bacterium *Photorhabdus luminescens*) can be used as a reporter system for detecting the phage lytic activity, which can be adapted to high-throughput plate readers. Metabolically active cells that constitutively express the luciferase operon produce a luminescent signal detectable by luminometry, while phage-induced cell lysis is manifested as a drop in luminescence (Meighen, 1991; Damron et al., 2013; Egido et al., 2023). In a recent study, the drop in luminescence was used to detect cell lysis for four different *Pseudomonas* phages (Egido et al., 2023).

### Detection of cell damage

An alternative approach to monitor cell lysis involves detecting damaged rather than intact metabolically active cells. These methods are primarily based on the disruption of bacterial membrane integrity during phage infection and the subsequent release of DNA, proteins, ATP, and ions into the medium.

The first method utilizes fluorescent dyes that stain nucleic acids but cannot penetrate intact membranes. Upon membrane damage, nucleic acids become accessible for dyes, resulting in fluorescence increase (Figure 3C). Initially developed to study enzyme- or antibiotic-induced cell degradation, this principle was recently adapted for phage infection tracking (Egido et al., 2023).

Two-Color viability assays use two fluorescent dyes that bind DNA but have different membrane permeability properties. One dye can penetrate cell membranes and stains all cells (e.g., Syto9, green fluorescence), while another dye cannot penetrate membranes and stains only cells with compromised membranes (e.g., propidium iodide, red fluorescence) (Emerson et al., 2017; Jassim and Griffiths, 2007). This allows to assess the relative numbers of dead and live bacteria in the sample (Decker, 2001; Tawakoli et al., 2013). These assays can be performed in bulk cell culture using fluorimetry (Latka and Drulis-Kawa, 2020; Jassim and Griffiths, 2007) and at the single-cell level using fluorescent microscopy or flow cytometry. For example, in a recent study a Live/Dead bacterial viability kit was used to explore the antibiofilm activity of *Klebsiella* phage KP34. The study determined the live-to-dead cell ratio of multidrug-resistant *K. pneumoniae* biofilms with and without phage treatment (Latka and Drulis-Kawa, 2020).

Tracking of the concentrations of small molecules and ions, released in the medium during cell lysis, provides another way to monitor lytic processes in cell culture. The increase in the extracellular ATP level (measured in enzymatic reactions) or the concentration of potassium ions (detected in real time using a K^+^-selective electrode) was successfully used to detect ongoing culture lysis (Daugelavičius et al., 2024; Ziedaite et al., 2005; Blasco et al., 1998; Liu et al., 2016; Dibrova et al., 2015). In a recent study on chemical antiphage defense, researchers ruled out the hypothesis that bacterial metabolites interfere with phage adsorption and DNA injection by demonstrating equivalent K^+^ release levels with and without this defense system (Kronheim et al., 2018). A recent study employed ATP quantification to assess the phage activity, by measuring the efficiency of phage-induced lysis in complex cultures alongside OD and H_2_S measurements (Santos et al., 2024).

Membrane potential changes is another indicator of cell lysis that can be tracked with lipophilic probes. Tetraphenylphosphonium (TPP^+^) is a small lipophilic cation that accumulates in membranes proportionally to the negative membrane potential maintained by live bacterial cells (Ziedaite et al., 2005). Membrane depolarization due to phage-induced lysis increases the extracellular concentration of TPP^+^, which can be monitored by selective electrodes. On the contrary, Phenyldicarbaundecaborane (PCB^−^) is an anion probe that binds to the membranes of metabolically inactive or damaged cells, serving as an early indicator of changes in the membrane permeability due to cell lysis (Daugelavičius et al., 2024).

The activity of enzymes released into the extracellular environment can also be used to detect cell lysis. It has been shown that detection of the adenylate kinase release is 10–100 times more sensitive than ATP detection, due to the enzymatic nature of the reaction (Blasco et al., 1998; Squirrell et al., 2002). Commercial kits for detecting the adenylate kinase activity have been adapted for high-throughput screening applications (Jacobs et al., 2013). For example, testing of the activity of leaked adenylate kinase was adapted for sensitive and precise identification of bacteria using a method that involves selective lysis of bacterial cells using phages (Blasco et al., 1998). Detection of other enzymes released from cells, such as lactate dehydrogenase or glucose-6-phosphate dehydrogenase, can also be used to detect cell damage, by measuring NADH production (Batchelor and Zhou, 2004; Kumar et al., 2018). Finally, release of reporter fluorescent proteins from infected cells also provides a proportional, sensitive lysis measure adaptable for high-throughput assays (Sharma et al., 2019).

### Measuring DNA amount and integrity

Analysis of phage and bacterial nucleic acids, involving DNA amplification and detection with sequence-specific probes, provides a powerful approach to study the dynamics of viral infection. Quantitative PCR (qPCR) employs fluorescent dyes that enhance emission upon binding to double-stranded DNA, enabling precise quantification of viral DNA titers (Abbott et al., 1988). The qPCR data can be directly used to calculate phage titers (PFU) using calibration curves (Luo et al., 2022; Projahn et al., 2020; Duyvejonck et al., 2019; Peng et al., 2018). Several qPCR variants are commonly used in phage research, as detailed below.

During infection, viral DNA replication can be tracked by qPCR sampling at multiple time points (Luo et al., 2022; Projahn et al., 2020; Šuster and Cör, 2023; Lisitskaya et al., 2023). However, standard qPCR cannot distinguish between DNA from infectious particles, damaged virions, or lysed infected cells (Duyvejonck et al., 2019; Girones et al., 2010). To address this, modified qPCR approaches that target only viable phages were developed.

To selectively detect live bacteria/infectious particles, sample pre-treatment can be used to eliminate non-viable DNA templates. Propidium monoazide (PMA) (Nocker et al., 2006) and its more efficient derivatives like ethidium monoazide (Chang et al., 2010) form covalent crosslinks with DNA upon light exposure and block polymerase reaction. PMA penetrates only damaged membranes or capsids and selectively neutralizes free nucleic acids and DNA from dead cells and damaged virions, while preserving signals from intact particles (Liu et al., 2014). This approach provides more accurate quantification of live bacteria and infectious viruses compared to conventional qPCR (Liu et al., 2014; Fittipaldi et al., 2010; Kim and Ko, 2012). By comparing PMA-treated samples with untreated controls, the method can also estimate the proportion of live versus dead cells or viruses in the population (Rudi et al., 2005). Alternative methods involve the use of DNA/RNA-degrading enzymes to eliminate extracellular nucleic acids prior to qPCR (Nuanualsuwan and Cliver, 2002; Otawa et al., 2008; Refardt, 2012). However, these methods can still overestimate the numbers of phages/bacteria by not accounting for objects with intact membranes/capsids but with damaged DNA.

The second approach is used to eliminate signals from damaged templates during qPCR. Long-amplicon qPCR (LA-qPCR) uses target DNA amplicons exceeding 400 base pairs, which reduces the detection of damaged DNA (Pecson et al., 2011; Ho et al., 2016). A combined approach utilizing both PMA and LA-qPCR (PMA-LA-qPCR) involves pre-treatement of the sample with PMA before amplification of long amplicons (Mclellan et al., 2016). This dual-filter methodology provides superior sensitivity for detecting viable pathogens, as it requires both membrane integrity (PMA exclusion) and DNA integrity (long amplicon amplification) (Mclellan et al., 2016; Banihashemi et al., 2012).

Droplet Digital Polymerase Chain Reaction (ddPCR) is a modification of qPCR that partitions the sample into thousands of nanoliter-sized droplets for amplification (Hindson et al., 2011). ddPCR provides superior sensitivity and accuracy compared to traditional qPCR, allowing for the calculation of the absolute number of template copies without the need for standard curves (Hindson et al., 2011; Hindson et al., 2013). This method has been used for monitoring the dynamics of virus and bacteria within infected population and analyzing the competition between two different viruses (Morella et al., 2018). The problem of background DNA signal from damaged cells and viruses can be addressed using the same methods as in the case of qPCR (Morella et al., 2018).

Southern blotting remains a valuable classic technique for detecting phage and host DNA during infection. This well-established method assesses phage DNA quantity and integrity using labeled DNA probes (Southern, 1975). Radioactive probes offer the highest sensitivity but fluorescent and chromogenic alternatives are also available. The method can be used to track phage replication dynamics and characterize host immune responses at the DNA level, in particular, by revealing which infection stages are inhibited and detecting large-scale DNA degradation (Rostøl et al., 2024; He et al., 2024; Patel et al., 2024).

To summarize, liquid culture approaches enable real-time monitoring of phage infection at the population level, often with superior throughput, scalability, and experimental flexibility in comparison with solid culture techniques. They remain indispensable for understanding population dynamics, phage growth parameters, and the evolution of phage-host interactions.

## Methods to study bacteriophages at single-cell level

**Figure 4:**
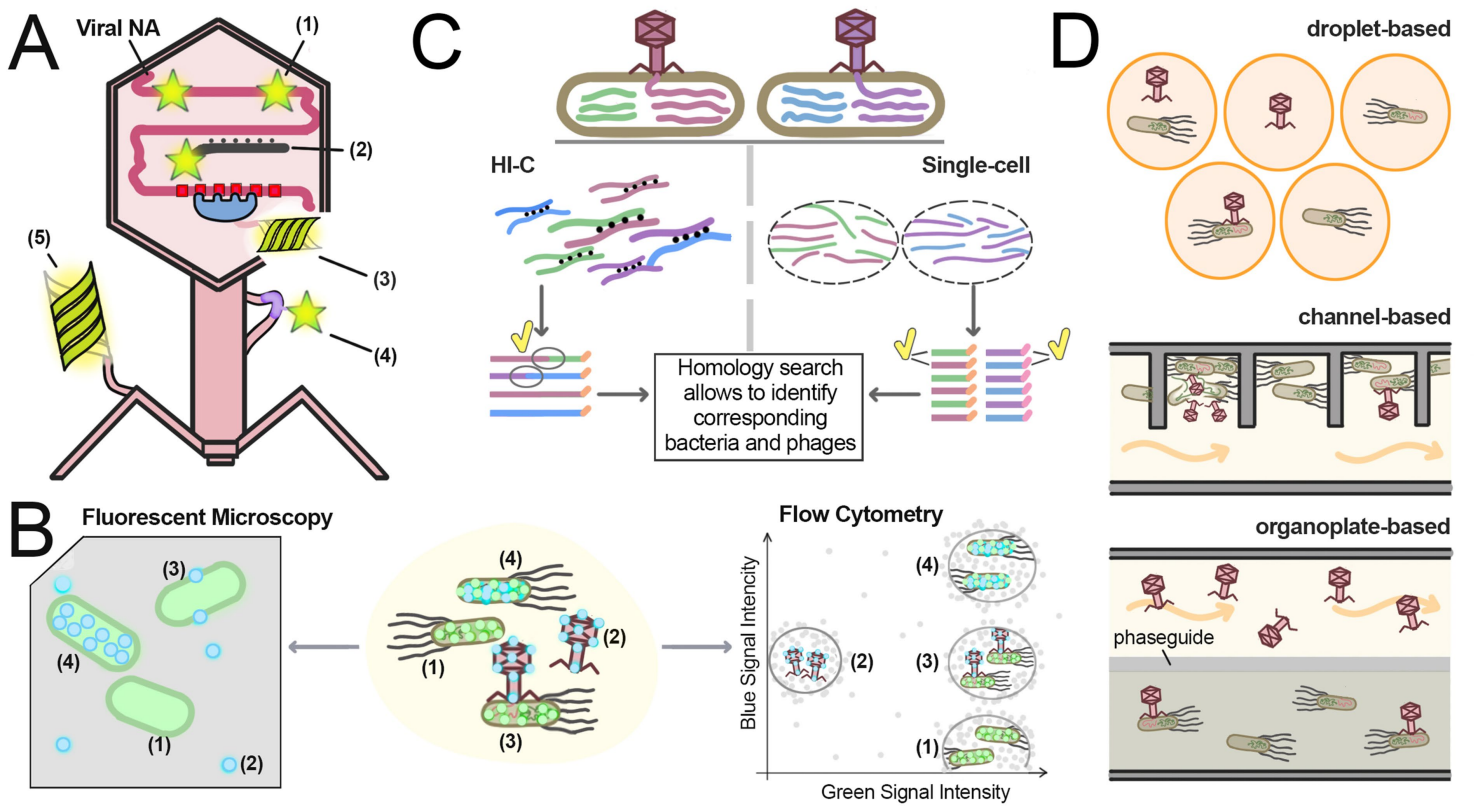
Single-cell techniques for studying phage infection. **(A)** Methods for labeling virions and phage molecules: (1) DNA-binding intercalating dyes; (2) Labeled nucleic acid probes targeting the phage genome; (3) Fluorescent proteins fused with DNA-binding proteins targeting specific sequences in phage DNA; (4) AHA-tags conjugated with fluorescent dyes; (5) Fluorescent proteins fused with phage capsid proteins. **(B)** Fluorescent microscopy and flow cytometry techniques. The sample shown in the center consists of labeled bacteria expressing fluorescent proteins (green) and capsid-labeled phage particles (light blue). Four cell sub-populations can be observed: (1) Free bacteria not infected with phages; (2) Free phage particles; (3) Bacteria in the early stages of infection, with phage capsids attached to their cell walls; (4) Bacteria in the late infection stages, actively producing fluorescent capsid proteins. Fluorescent microscopy (left) provides direct observation of sample entities, offering more detailed but slower analysis compared to flow cytometry. Flow cytometry (right) quickly measures parameters of individual cells in the sample, presenting the data as charts showing the intensity of different signals for each entity in the sample. The graph contains four regions corresponding to each sub-population. Notably, most fluorescent microscopes and flow cytometers cannot effectively analyze free phage particles due to the low intensity of their signals. **(C)** Hi-C and single-cell sequencing approaches for identifying phage-host linkages. These protocols analyze samples containing mixtures of phage-infected bacteria. Two different bacteria infected with two different viruses, distinguished by the coloring of their DNA, are shown on the top. In the Hi-C protocol (left), the samples are treated with crosslinking agents before phage extraction. Crosslinks (black dots) connect nucleic acid molecules, allowing linkage of different DNA fragments within the same cell, including host and phage DNA. All DNA fragments from the same cell share the same barcode (orange extensions in reads). Sequencing of these linked fragments reveals host-phage associations, as fraction of reads contains both phage and bacterial DNA (ovals with a yellow tick). In single-cell sequencing protocol (right), individual bacteria are separated, and DNA fragment from each one is uniquely labeled with different barcodes (orange and pink extensions in reads). Homology searches then assign phage and bacteria to taxonomic groups. Phage-host linkages are established by detecting either phage and bacterial sequences in the same read (Hi-C) or shared barcodes (single-cell sequencing). **(D)** Different microfluidics designs. In droplet-based microfluidics bacteria and phages are sequestered in small droplets. In channel-based microfluidics liquid media constantly flows (orange arrows) through channels, replenishing nutrients; bacteria and phages reside in chambers connected to the main stream. In organoplate-based microfluidics bacteria reside in semi-solid media while phages diffuse from liquid media through the phaseguide.

The main limitation of the population-based methods discussed so far is that the data obtained represent an average across many cells, while bacterial population can be highly heterogeneous (Eisenstark, 1967; Lidstrom and Konopka, 2010; Kümmerli and Frank, 2023). The 21st century brought us to a new era of single-cell analyses, including high-resolution microscopy, flow cytometry, microfluidics and high-throughput sequencing techniques, and allowed researchers to investigate cell-phage interactions both under laboratory conditions and in natural populations (Martínez Martínez et al., 2020; Putzeys et al., 2024). These methods made possible visualization of all infection steps, showing how individual viruses adsorb host bacteria, enter the cells, shift between life cycle stages, and eventually exit the host. Moreover, they enabled the study of interactions between uncultivated bacteria and their phages in bacterial communities in natural habitats, greatly expanding our understanding of microbial ecology beyond laboratory model systems. This section explores techniques that can yield detailed mechanistic insights into the infection process at the single-cell/single-phage level, summarized in Figure 4.

### Light and electron microscopy in phage research

The first device for observing microscopic objects, the microscope (from Greek *micros*, small, and *skopeo*, to look at), was invented in 1590 by Hans and Zacharias Janssen and consisted of several lenses placed in a tube (Knight, 2015). However, the true exploration of the microscopic world began with the efforts of Robert Hooke and Antonie van Leeuwenhoek, whose observations of microscopic objects were widely published, leading to the widespread adoption of microscopy (Hooke, 1665; van Leeuwenhoek, 1798). Since the first half of the 20th century, microscopy has been widely used to study viral infections. The challenge is that many bacteria are transparent and refract light in a manner similar to their surrounding medium, which complicates their visualization. Contrast-enhancing techniques allow researchers to observe living cells in real time, whereas dyes often require cell fixation (Barer, 1974).

Phase-contrast microscopy, one of the most widely used contrast enhancement techniques in phage research, was invented by Zernike (1935). This method converts small differences in the phase of light waves into changes in intensity, allowing the visualization of unstained, transparent samples, including bacterial cells, in real time without staining (Rohde, 2011). It made possible to visualize phage-induced lysis of individual bacterial cells in real-time for the first time (Rice et al., 1954) and continues to be actively used in phage research (Wu et al., 2021; Dennehy and Wang, 2011). Modern equipment allows the combination of phase-contrast microscopy with fluorescence microscopy. However, it is still a less informative method compared to other microscopic techniques.

Electron microscopy is another powerful tool for studying unlabeled objects. The key advantage of this method is its far greater resolution and magnification than light microscopy. In 1933, Ernst Ruska developed the first prototype of an electron microscope (Lambert and Mulvey, 1996). The first electron microscopy images of bacteriophages were obtained by 1940 (Ackermann, 2011). Ruska’s images depicted a bacterium surrounded by bacteriophages, while Pfankuch and Kausche captured images of individual virions. Further advances in electron microscopy made it possible to visualize all stages of the infection cycle from viral entry to progeny release. High-quality electron micrographs can reveal viral adsorption, injection of viral DNA accompanied by virion emptying and its conformational changes, virions assembly, and cell lysis (Simon and Anderson, 1967; Böhm et al., 2001; Marling, 1949). However, the main disadvantages of electron microscopy include the need for expensive equipment and the invasive nature of sample preparation. For a comprehensive review of the use of electron microscopy in phage research, see Ackermann (2012).

### Phage and bacteria labeling and fluorescence microscopy

Labeling of viral and bacterial components can be used to explore physical interactions between cells and viruses, identify specific cell types, visualize phage replication, cell division and other physiological changes. These methods usually rely on fluorescent proteins, membrane dyes or tags/probes to nucleic acids for visualization of specific cellular and viral structures (Figure 4A). Fluorescence microscopy has become a vital technique in bacterial cell biology, allowing highly specific and sensitive visualization of labeled bacterial and viral components, analysis of their spatial distribution and dynamics (Figure 4B) (Yao and Carballido-López, 2014). Furthermore, super-resolution techniques have provided unprecedented details of phage-bacteria interactions and are one of the most prominent direction in modern virus studies (Kiss et al., 2020; Robb, 2022).

Intercalating dyes that alter their fluorescence upon binding to nucleic acids are one of the simplest methods of labeling. Some dyes can integrate into viral nucleic acids without disrupting the virion integrity or infectivity (Eriksson et al., 2007; Mosier-Boss et al., 2003; Brussaard, 2009). Early studies demonstrated that intercalating dyes can be used for detection of viral particles without electron microscopy (Hennes and Suttle, 1995; Hennes et al., 1995). For instance, Oxazole Yellow staining revealed far higher phage concentration in environmental water samples than previously estimated and enabled visualization of bacteria with adsorbed phage (Hennes and Suttle, 1995). The discovery of more sensitive dyes, like SYBR Gold (Tuma et al., 1999), which can stain RNA, ssDNA, and dsDNA phages, further advanced this approach. For example, SYBR Gold-labeled phage P22 was used for identification of its host *S. typhimurium* in mixed cultures (Mosier-Boss et al., 2003). Dye-labeled viruses were also used as probes for quantification of bacterial hosts in water samples (Hennes et al., 1995) and for flow-cytometry sorting of phage-tagged bacteria (Deng et al., 2012). In a landmark 2012 study, researchers used direct staining of phage DNA with a cyanine dye to track DNA ejection from phage virion and its entry inside bacterial cells by fluorescent microscopy (Van Valen et al., 2012).

As discussed above, staining of cellular DNA with intercalating dyes can also be used for distinguishing live and dead cells during infection, since the permeability of the membrane to different dyes varies (Yoon et al., 2021). Combining fluorescence microscopy with two-color cell viability staining allows real-time analysis of phage infection. Phage-induced lysis results in an increase in dead-cell fluorescence, which can be detected by bulk fluorescence measurements or by microscopy (Liu et al., 2016; O’Flaherty et al., 2005; O’Flynn et al., 2007). This assay can also be adapted for staining entire colonies in solid cultures, revealing dead-cells and irregular morphology in virus-infected colonies (O’Flynn et al., 2007).

A key limitation of the majority of dye-based methods is the lack of selectivity since these dyes stain all nucleic acids in the sample nonspecifically. Hybridization *in situ* allows for the spatial localization of specific nucleic acid sequences within a sample using complementary labeled nucleic acid probes (Langer-Safer et al., 1982). In FISH (Fluorescence *In Situ* Hybridization), the fluorescent molecule can either be directly fused to the probe, or the probe can be conjugated with biotin for avidin-based detection (Langer-Safer et al., 1982). Unlike nonspecific dyes, FISH labels nucleic acids selectively, but requires sample fixation, permeabilization, and hybridization, complicating kinetic analysis (Huber et al., 2018). Nevertheless, FISH was used to track MS2 phage infection in samples prepared at different times of infection (Harb et al., 2020). Despite their high specificity, FISH-based methods for phage research have limitations including complex sample preparation with potential artifacts, low quantitative accuracy, and challenges in detecting low-abundance targets.

Variations of FISH methods allow linking phages to their hosts by detecting co-localization of phage and bacterial DNA. GeneFISH combines probes that target rRNA and genes of interest to analyze the distribution of specific genes within natural microbial populations without cultivation (Moraru et al., 2010). PhageFISH is an adaptation of this method for phage research that relies on simultaneous labeling of bacterial 16S rRNA and phage DNA and can be used for: (1) detection of intracellular and extracellular viral DNA, (2) quantification of the ratio of infected to noninfected cells, (3) semi-quantitative predictions of the phage number per cell, and (4) establishment of phage-host linkages in mixed cultures (Allers et al., 2013). The development of single-cell transcriptomic methods has led to the development Single Molecule FISH (smFISH), which was applied to study viral infection in phytoplankton *Emiliania huxleyi* using dozens short fluorescent probes specific to mRNA of single algae or viral genes (Vincent et al., 2021).

Viral and cellular DNA can also be visualized using fluorescent proteins fused to sequence-specific DNA binding domains, expressed in the host bacteria and recognizing engineered binding sites in the phage or host genome (e.g., ParB-mCherry targeting parS sites or other; Figure 4A). This allows labeling of viral DNA and visualization of phage entry and replication during infection (Bulssico et al., 2023; Trinh et al., 2020; Tal et al., 2014; Zhang et al., 2021). Selective labeling of DNA with fluorescent proteins can be used to detect active viral DNA replication within infected cells (Dang et al., 2015), as well as to measure the number of bacteriophage copies per cell (Harb et al., 2020).

Bacteriophages can also be visualized using fluorescent tags fused to various viral components. The most common approach involves using of host bacterial strains with plasmids encoding a viral capsid protein fused to a fluorescent protein (Bulssico et al., 2023; Trinh et al., 2020; Zhang et al., 2021). Induction of the chimeric protein expression during viral replication leads to incorporation of fluorescent proteins into the viral capsid alongside the normal capsid proteins. Using of labeled bacteriophage virions allows direct identification of infected cells, analysis of viral adsorption, and measurement of MOI for individual cells. The assembly and release of virions during infection can also be directly observed in cells expressing fluorescently tagged viral capsid proteins (Bulssico et al., 2023; Trinh et al., 2020; Zhang et al., 2021; Zeng et al., 2010). However, modification of capsid proteins can disrupt virion structure and affect phage infection, requiring careful design of fluorescence reporters.

Another common approach is the creation of reporter viruses producing fluorescent proteins upon invading the cell, thereby allowing identification of infected cells (Bulssico et al., 2023). The disadvantages are the need to obtain modified viruses and long maturation periods for many fluorescent proteins, unsuitable for rapidly replicating lytic viruses. Fluorescent proteins can also be cloned under the control of native viral or bacterial promoters to detect various physiological states of the infected cell, such as SOS response, or track various stages of the lytic cycle in individual cells (Bulssico et al., 2023; Zeng et al., 2010; Amir et al., 2007; Bednarz et al., 2014; Baek et al., 2003).

Bioorthogonal labeling (BioOrthogonal Non-Canonical Amino acid Tagging, BONCAT) allows to incorporate non-canonical amino acids, such as 4-azido-L-homoalanine (AHA), into phage proteins during virion assembly *in vivo*, which can then be conjugated with fluorophores or biotin by click chemistry (Hellwig et al., 2024). Subsequent fluorescence microscopy, flow cytometry or avidin agarose pull-down enable to visualize and sort host cells with viral particles (Hellwig et al., 2024). Recently, a similar technique with improved labeling efficiency, employing a threonine analog (THReOnine-derived Non-Canonical Amino acid Tagging, THRONCAT), was introduced but it remains untested in phages (Ignacio et al., 2023). The limitations of these methods include potential interference of such modifications with virion assembly and variable labeling efficiencies across phage species.

### Flow cytometry

The Flow Cytometry (FC) technique traces its origins to the Moldavan’s (1934) approach for cell counting, which aligned cells in a fluid stream for detection via a microscope-photoelectric apparatus. Modern instruments rely on fluorescence detection, allowing quantification of cells labeled with antibodies, DNA- and RNA-specific dyes, viability markers, or fluorescent proteins (McKinnon, 2018). One of the key strengths of flow cytometry is its ability to rapidly analyze large numbers of cells while simultaneously measuring multiple parameters (McKinnon, 2018). Instead of producing images, FC provides numerical data on the prevalence of cell subpopulations. This allows for rapid and precise determination of the proportions of bacteria in different states (Lindström, 2012; O’Connor, 1996) (Figure 4B).

FC is a powerful alternative to traditional methods for quantifying bacterial populations in endpoint infection assays. Cell staining with intercalating dyes allows direct cell quantification after viral infection, bypassing the need for CFU counting but potentially including non-viable cells (Verthé and Verstraete, 2006). When combined with two-dye viability staining, this approach provides quantitative assessment of phage-mediated killing across populations (Verthé and Verstraete, 2006; Pires and Melo, 2018). Synchronized infection experiments with timed sampling make it possible to track the accumulation of dead cells and detect the point of culture collapse (Melo et al., 2018; Melo et al., 2022; Silva and Melo, 2024; Ameh et al., 2020; Ameh et al., 2018).

FC can be used to count phages when no cultivable host is available for plaque counting (Brussaard, 2009; Oliveira et al., 2017). Pre-staining of virions with intercalating dyes or fluorescent proteins allows for the measurement of the total number of viral particles in the sample using FC. However, it requires ultra-sensitive detection instruments as viral DNA is small compared to the genomes of most bacteria, resulting in low fluorescence intensity (Brussaard, 2009; Oliveira et al., 2017). Unlabeled bacteriophages can be counted by measurements of light scattering, available in most cytometers (Ma et al., 2016). A flow cytometry-based method for measuring the phage adsorption rate has also been proposed (Zemb et al., 2013).

While flow cytometers typically discard individual cells during analysis, fluorescence-activated cell sorting (FACS) separates cells into fractions based on predefined parameters and can be used for isolation of virus-targeted bacteria (Telford, 2023). For example, cell sorting can be applied for isolation of uncultured host bacteria and their phages from environmental samples. In Phage Receptor-binding proteins-Activated Cell Sorting (PhRACS), environmental bacteria are tagged with fluorescently labeled phage receptor-binding proteins, allowing their extraction from mixed populations by FACS. A similar technique employs fluorescently-labeled cell wall-binding domains of viral endolysin proteins for isolation of specific bacterial hosts using FACS, followed by His-tag based separation of bacteria on magnetic beads (Hosokawa et al., 2023). Since phage proteins can potentially bind to non-host cells, identified phage-bacteria pairs need to be verified using other methods. Cell sorting has also been employed for the selective isolation of virus-infected bacteria, using fluorescently tagged phages to distinguish infected and uninfected cells (Deng et al., 2012; Unterer et al., 2023).

In comparison with fluorescence microscopy, FC provides high-throughput quantitative data on subpopulation distributions and allows faster sample processing (Lindström, 2012; O’Connor, 1996). At the same time, conventional flow cytometry cannot track individual cells over time or provide spatial information about intracellular organization. Combinations of fluorescence microscopy and flow cytometry can help to overcome the limitations of both techniques (Godfrey et al., 2005). For example, recently developed Imaging Flow Cytometry method allows to rapidly take images of individual cells while they are passing through the detector (Rees et al., 2022). In summary, diverse labeling techniques for nucleic acids and proteins enable single-molecule and single-cell tracking of phages and bacteria through advanced imaging and sorting technologies.

### Microfluidics-based techniques

Microfluidics is a relatively new field that encompasses a wide range of devices and techniques designed to perform experiments on a miniaturized scale, using small volumes of liquids and particles. For an introduction to microfluidics and its applications in bacterial research, we recommend the reviews by Whitesides (2006) and Pérez-Rodríguez et al. (2022). There are several microfluidics designs often employed in phage research. Droplet microfluidics enables analysis of individual phages and bacteria by encapsulating them in picoliter- to nanoliter-scale droplets. Channel microfluidics uses devices consisting of microscale channels with dimensions in submillimeter range with controlled fluid flow, where bacteria can be grown (Whitesides, 2006). Compared to traditional techniques, microfluidic devices enable fine-tuned manipulation of environmental parameters, including chemical gradients, temperature, oxygen levels, and fluid flow (Pérez-Rodríguez et al., 2022). Microfluidic systems are usually integrated with fluorescence microscopy to observe bacteria and phages in real time using the labeling techniques described above. Several microfluidic techniques that have been used to analyze phage infection dynamics are described below (Figure 4D).

One channel-based microfluidic system employs a microfluidic culture plate (“Organoplate”) with 96 small chambers with thin glass bottom for direct microscopic observation. Each of the chambers contains two parallel channels separated by a thin ridge called a phaseguide which allows partial diffusion between chambers (Mabrouk et al., 2023). Bacteria expressing fluorescent proteins are grown on semi-solid agar medium in one of the channels, while the bacteriophage sample is passed in liquid medium through the second channel. Designed to isolate and characterize new phages, this platform monitors bacteria-phage interactions in real time and can capture bacterial lysis, phage resistance, and morphological changes during infection (Mabrouk et al., 2023). With its high-throughput capabilities, it allows simultaneous study of multiple combinations of bacteria and phages, but requires specific materials and equipment.

Droplet-based microfluidics has also been successfully used to isolate and detect phages (Hoshino et al., 2023). In this approach, a droplet generator produces miniature droplets, each acting as an isolated microenvironment for phage-host co-cultivation. Each droplet harbors labeled host bacteria infected with phages and a fluorescent dye that binds to newly synthesized phage DNA. An increase in the fluorescence signal identifies droplets with successful phage propagation. Such droplets can then be sorted into a 96-well plate for subsequent phage isolation. By analyzing thousands of droplets in parallel, this method achieves high-throughput phage screening and phage detection in environmental samples (Hoshino et al., 2023). A related droplet-based method allows prolonged imaging of phage-bacteria interactions, by immobilizing droplets in miniature wells for time-lapse fluorescence microscopy (Nikolic et al., 2023). The use of GFP-labeled bacteria allows quantification of population dynamics, including growth rates, doubling times, and lysis events, providing high-resolution, kinetic data on phage-mediated bacterial killing. Droplet-based microfluidics has potential disadvantages including droplet coalescence, biased encapsulation efficiency favoring certain phage-host pairs, and limited scalability due to the technical complexity of droplet generation and analysis.

A recently described droplet-based microfluidic technique allows to estimate phage concentration using a ‘digital phage SlipChip’ device and principles analogous to ddPCR (Li et al., 2023). Here, nanoliter droplets are used to compartmentalize phages and bacteria, and bacterial growth is monitored by light microscopy. Droplets devoid of growth indicate phage-mediated lysis, while growing cultures suggest phage absence. Applying Poisson statistics to these outcomes yields an accurate phage count. Analogous to ddPCR, each droplet functions as an independent assay, detecting only infectious phages. A recent preprint by Givelet et al. (2025) also highlights the advantages of using droplet-based microfluidics for phage quantification. This method allows for precise control of MOI and exposure time, which is essential for accurate determination of lysis events.

Tadmor et al. (2011) developed a microfluidic ddPCR approach to identify virus-host pairs directly from environmental samples. Diluted samples are loaded into a device designed to isolate single bacterial cells per chamber. Amplification of bacterial and viral gene markers using ddPCR reveals phage-host pairs when both signals are detected in the same chamber. The Emulsion Paired Isolation-Concatenation PCR (epicPCR) can also be used for phage-host pair identification, by creating chimeric bacterial-viral amplicons if both target genes are present in the same droplet, which can then be sequenced (Sakowski et al., 2021). Both ddPCR-microfluidics and EpicPCR can process thousands of individual droplets simultaneously, allowing researchers to capture and analyze numerous interactions in a single experiment without the need for cultivation. The limitation of this approach is the lack of universal phage genes for amplification, but the method works well for specific phage groups.

In summary, microfluidics enables single-cell analysis of phage-bacteria interactions and can be applied for analysis of uncultivated bacteria from environmental samples, but demands specialized equipment and expertise. It is typically coupled with imaging or sequencing platforms for visualization and identification of interacting bacteria and phages.

### NGS methods in phage research

The use of next generation sequencing (NGS) methods has dramatically expanded our understanding of bacteriophage diversity in the environment, including uncultivated phages and host bacteria. The main disadvantage of these approaches is the destruction of cells during library preparation. But they are widely used in modern biology, including phage research. For example, a large-scale human microbiota study identified over 100,000 bacteriophages through database mapping (Camarillo-Guerrero et al., 2021). Metagenomic analysis of viromes is performed using several available tools including vRhyme (Kieft et al., 2022), PHAMB (Johansen et al., 2022), COBRA (Chen and Banfield, 2024) and ViromeFlowX (Wang R. H. et al., 2024). PhageScope, a curated database, integrates diverse phage sequences with functional annotations (Wang R. H. et al., 2024).

The ability to extract viromes from metagenomic data has uncovered links between microbiota, phages and human health, with disease prevalence often correlated with specific microbiome profiles (Hou et al., 2022). It was shown that viral diversity varies with age (Zeng et al., 2024) and declines in disease state (Zhang and Wang, 2023; Kirk et al., 2024). For instance, a recently described phage order *Ca.* Heliusvirales has been linked to urban-associated conditions like metabolic syndrome, diabetes, and inflammatory bowel disease (de Jonge et al., 2024; de Jonge et al., 2022). Mouse models have confirmed inflammatory bowel disease (IBD)-virome associations (Tian et al., 2024), and gut virome alterations have been implicated in type 2 diabetes (Fan et al., 2023) and asthma (Leal Rodríguez et al., 2024).

Despite huge progress in microbiome and virome sequencing, phage-bacteria interactions *in vivo* remain poorly understood and phage-host correlations are challenging to establish. Analysis of the spacer content of CRISPR cassettes can be used to link phages to hosts; for example, such approach revealed striking contrasts between rumen and human gut viromes (Yan et al., 2023). However, its throughput is limited and many phages cannot be matched to any CRISPR sequences. A comprehensive review of bioinformatics tools used for phage host prediction was recently published by Clément Coclet and Simon Roux (Coclet and Roux, 2021).

Recently developed NGS approaches can provide data on virus-host interactions at the single-cell level (Figure 4C). A high-throughput chromosome conformation capture (Hi-C) method, which uses formaldehyde crosslinks prior to sequencing, significantly enhances metagenome analysis. Crosslinking bacterial and phage DNA within cells enables phage-host pairing during library preparation, a feat impossible with conventional NGS. For example, combining long-read sequencing with Hi-C analysis identified nearly 200 novel virus-host pairs in the rumen microbiome (Leal Rodríguez et al., 2024). Similar approach uncovered hosts of crass-like phage in the human gut microbiome (Marbouty et al., 2021). In soil, Hi-C coupled with metagenomic DNA/RNA sequencing identified some virus-host pairs and showed that drying induces lysogeny and reduces viral transcription (Wu et al., 2023). It was shown that Hi-C-based bioinformatics tools, such as ViralCC, outperform other phage binning methods in efficiency (Du et al., 2023). However, Hi-C-based NGS methods exhibit decreased sensitivity in low-biomass or complex microbiomes due to insufficient crosslinking events between rare phage-host pairs and elevated noise-to-signal ratios due to nonspecific DNA ligation.

Single-cell RNA sequencing (scRNA-seq) can elucidate phage-bacteria connections without the crosslinking step, by analyzing the transcriptomes of single cells. In this approach, the presence of bacterial and phage transcripts in the same cell is interpreted as a sign of successful infection. However, these methods lose some information during sample preparation, and miss phages that may remain in an inactive form. SmRNA-seq was successfully applied for testing bacterial susceptibility to phage infection (Wang B. et al., 2023), uncovering phage-host pairs in the human microbiome (Shen et al., 2024), and studying mixed bacterial communities and prophage induction (Wang B. et al., 2023).

In summary, single-cell approaches make possible high-resolution observation of cell-phage interactions, allowing to reveal population heterogeneity and extending these studies to uncultivated microbes.

## Conclusion

In the 20th century, phage research relied mainly on cultivated bacteria and population-level assays. While traditional bulk solid/liquid culture methods remain widespread due to their simplicity and scalability, they ignore cellular heterogeneity by averaging data across populations. The 21st century has introduced single-cell and high-throughput techniques, revealing phage-bacteria interactions in unprecedented detail in both laboratory and environmental samples, including for uncultivated hosts. Modern phage biology thrives at the intersection of population-scale and single-cell methods, and brings together fundamental and applied research. Classical methods are convenient to use in routine experiments, due to low financial cost and no need in bioinformatic data processing. Solid media methods allow for quick identification and calculation of phage particles. In liquid culture, additional modifications can be made and lysis can be detected more efficiently. Working with single cells requires specialized skills and equipment, but provides high-resolution data unavailable from classic approaches. Most modern studies on molecular mechanisms of infection, analysis of microbial communities and optimization of phage cocktail therapies rely on single-cell techniques. Today, fluorescence microscopy, flow cytometry, microfluidics and NGS, often used in combinations, are indispensable for high-throughput, quantitative phage research. Combining multiple approaches can elucidate the molecular mechanisms of the infection processes and expand our understanding of phage-bacteria interactions across ecosystems.

A promising direction in future phage research is the application of CRISPR-Cas tools for manipulating phage genomes, allowing researchers to study the infection mechanisms (Mahler et al., 2023) and control phage gene expression (Bergmiller, 2025). CRISPR-Cas cassettes with spacers targeting bacterial genomes can be integrated into phages to effectively eliminate bacterial cells, even in biofilms, in phage therapy (Gencay et al., 2024). The CRISPR-dCas13 systems targeting specific RNA transcripts have emerged as a powerful tool for studying the phage gene function (Adler et al., 2025). The SEC-MS method reveals dynamic changes in the proteome during infection (Fossati et al., 2023).

The future of phage research also lies in integrating AI and machine learning (ML) algorithms with experimental data. AI is currently being used for image analysis and prediction of host-phage pairs, optimizing phage therapy cocktail compositions, and facilitating the design of synthetic phages with enhanced therapeutic properties (Boeckaerts et al., 2024; Gaborieau et al., 2024; Doud et al., 2025). A large number of open-source software tools are available for phage research. For example, Serratus accelerates identification of phages in large datasets (Edgar et al., 2022). ML models improve host prediction (Boeckaerts et al., 2024; Shang and Sun, 2021) and the viral lifecycle (Zhang et al., 2024), simplifying experimental design. In the future, AI-driven phage engineering may help to fight bacterial infections and overcome antibiotic resistance.

Undoubtedly, the use of modern technologies in the field of phage biology, together with classical approaches, will significantly enrich this field.

## Glossary

Bacterial lawn: A confluent, homogeneous layer of bacteria on agar.
Burst size (Kropinski, 2018): The number of infectious viral particles synthesized by a single infected cell.
Colony-Forming Units (CFU): A quantitative measure of viable, culturable bacteria in a sample.
Latent period (Kropinski, 2018): Time from phage adsorption to host lysis.
Lysogenic Cycle (Weinbauer, 2004): A viral reproductive cycle in which the phage genome integrates into the host cell’s chromosome as a prophage and replicates passively along with the host DNA.
Lytic Cycle (Weinbauer, 2004): Active phage propagation state ending by cell lysis and the release of new viral particles.
Nonhost (resistant) bacteria: Strains insensitive to infection by a specific phage.
Multiplicity of Infection (MOI) (Abedon, 2016): The ratio of infecting phage particles to susceptible bacteria.
Phage-mediated bacterial lysis (Young and Wang, 2005): Bacterial cell rupture followed by the release of phage progeny.
Plaque-Forming Unit (PFU) (Kropinski, 2018): A quantitative measure of viable, plaque-forming phages in a sample; corresponds to an infectious phage particle that can form a plaque on bacterial lawn.
Prophage (Bruce et al., 2021): Phage DNA present within the infected host in the state of lysogeny. Prophages can exist either integrated into the host genome or as plasmids.
Susceptible or indicator bacteria (Abedon and Yin, 2009): Bacterial strains permitting productive phage replication.
Soft agar (Adams, 1959): A semi-solid agar medium (agar percentage: 0.3–0.7%) that facilitates phage diffusion and plaque formation.
Temperate Phages (Gandon, 2016): Bacteriophages that can alternate between the lytic and lysogenic life cycles.
Viral adsorption (Kropinski, 2009): Phage attachment to bacterial receptors, followed by the injection of viral DNA into the bacterium.
Viral infectivity (Black, 1993): The ability of a virus to enter cells and replicate productively.
Viral plaques (Abedon and Yin, 2009): Visible, clear zones in a bacterial lawn caused by phage-mediated lysis. Each plaque typically arises from a single infectious particle.
Virion (Adams, 1959): A viral particle consisting of genetic material (either DNA or RNA) and a protein coat.
